# Thematic analysis comparing stressors for pediatric residents and subspecialty fellows at a large children’s hospital

**DOI:** 10.1080/07853890.2022.2148731

**Published:** 2022-11-21

**Authors:** Nimisha Bajaj, Suzanne M. Reed

**Affiliations:** Department of Pediatrics, The Ohio State University College of Medicine, Nationwide Children’s Hospital, Columbus, OH, USA

**Keywords:** Pediatrics, residency, fellowship, stressors, self-reported, well-being

## Abstract

**Introduction:**

Pediatric residents and subspecialty fellows experience a significant number of stressors during training, but they have rarely been self-reported or compared between groups. This qualitative study aimed to identify and compare themes of stressors experienced by pediatric residents and subspecialty fellows at a single large children’s hospital.

**Materials and methods:**

Using an open-ended survey at single time point for each group, we asked residents and fellows to list the stressors they face in training. The survey data was iteratively analyzed using thematic analysis then quantified by its frequency in each group and compared using a chi-square distribution or a Fisher’s exact test, as appropriate.

**Results:**

Twenty-eight of 159 residents (18%) and 38 of 180 fellows (21%) answered the survey question, and an average of 2.8 stressors were identified by each resident and fellow. Two major themes and five major subthemes were shared between both groups. The theme *Stressors at Home* included the subthemes *Difficulty Maintaining Overall Health* and *External Stressors*. The theme *Stressors at Work* encompassed the subthemes *Clinical Stressors Innate to Patient Care, Demanding Workload and Schedule,* and *Stressors Related to Culture of Work Environment*. Within the subthemes, there were differences in categories of stressors between the groups. While there was no statistically significant difference in the distribution of themes, subthemes, or categories of stressors mentioned between groups, in general residents identified stressors associated with lack of autonomy and control whereas fellows focused on clinical uncertainty and complex situations.

**Conclusions:**

While residents and fellows shared similar themes and subthemes for stressors, there was variability between individual categories. This study identified individual self-reported stressors that can be used by programs to design interventions to improve trainee well-being, but it also implies that programmatic support at different levels of training should be tailored to the target group.

**KEY MESSAGE**
- At our hospital, we found that some self-reported stressors facing pediatric residents and fellows were common and some unique.
- Stressors included those that can be eliminated or diminished as well as those that cannot.
- With knowledge that these disparities exist, training programs should use unique strategies to provide support for the two groups and their stressors.

## Introduction

Burnout and depression are rampant in pediatric trainees, with 31–75% of pediatric residents meeting criteria for burnout and 10–25% for depression [1–4]. Residents with depression, which is associated with burnout, also show higher rates of cognitive impairment, difficulty concentrating at work, and a six-fold increase in medical errors [2,3]. Studies show that 13.6% of pediatric residents regret their career choice [4], and depression and burnout are associated with decreased job satisfaction [1]. Certain pediatric subspecialty fellows have been shown to have similar levels of burnout to residents (30–39%) [5,6], correlating with worse outcomes on scales assessing empowerment, patient-centeredness, self-assessment in humanism, and satisfaction with training [6]. In general, residents and fellows have higher rates of burnout, depression, and suicidality than age-matched college graduates [7].

While rates of burnout and lack of job satisfaction in pediatrics compare to those of other specialties [4], pediatricians are also valued for their compassion, altruism, and perfectionism, all traits that predispose them to burnout [8]. Additionally, female physicians have a higher rate of burnout than males [4], and as of 2015, approximately 75% of pediatric trainees were female [9].

In 2017, the Accreditation Council for Graduate Medical Education (ACGME) updated its common program requirements for fellowship to include language that compels programs to promote fellow well-being, stating, ‘psychological, emotional, and physical well-being are critical in the development of the competent, caring, and resilient physician’ [10]. Residency program requirements were updated the same year [11]. Pediatric residency educational leaders have been publishing data since 1991 on wellness initiatives. Such interventions have led to decreased burnout and stress and increased resilience and mindfulness and include workshops for stress management [12], stress reduction and enhanced communication [13], and mind-body skills training [14]. Pediatric residency leaders even established the Pediatric Residency Burnout-Resilience Study Consortium, a collaborative of over 40 pediatric training programs who study epidemiology of and mitigation strategies for burnout in pediatric residents [15]. One study from this group found more than 97% of pediatric residency programs had active or passive well-being interventions for their residents [16].

Despite the work being done in pediatric residency programs to promote trainee well-being, less has been done in pediatric subspecialty fellowship programs. Recently, studies examining narrative medicine in critical care fellows [17] and humanism and professionalism training in hematology oncology fellows [18] have shown small improvements in burnout, depression, secondary trauma scores, and work-life balance.

Interventions to promote trainee wellness should take into account the stressors leading them to be unwell. While residents and fellows are viewed similarly, they have unique training experiences, including different work hours, academic requirements, and level of responsibility. No studies have identified self-reported stressors for pediatric residents; they have only been identified by program directors [19] or by collecting objective data such as work hours and demographics and comparing them to burnout data [4,20,21]. Only a few studies have queried pediatric subspecialty fellows about their stressors [5,22,23], but all of these studies focused on specific subspecialties rather than pediatric fellows more generally.

There is a gap in the literature in pediatric residents and most pediatric subspecialty fellows directly reporting their stressors during training. In this commentary, we reported our study results comparing broader themes of stressors identified by residents and fellows themselves at a single large children’s hospital, so that programs may use these themes to design unique interventions for fellows and residents to promote their well-being.

## Materials and methods

This was a cross-sectional study evaluating stressors for residents and fellows at individual time points. Our data was collected as part of two separate needs assessment surveys sent to fellows and residents for the purpose of designing two separate wellness interventions. This work was approved by the Nationwide Children’s Hospital Institutional Review Board under STUDY00001270 and STUDY00001983. Because there was minimal risk to this study with no identifying information, in order to encourage participation, consent was implied with participation in the survey.

### Study recruitment

#### Residents

All pediatrics and medicine-pediatrics residents at Nationwide Children’s Hospital were invited to complete the survey. They were recruited via email and given three weeks in July to August 2020 to complete a six-question survey using SurveyMonkey online survey software.

#### Fellows

All fellows at Nationwide Children’s Hospital represented by the Fellow Resident Advisory Council were recruited to complete the survey. This included predominantly pediatric subspecialty fellows, but also a few surgical, anesthesiology, radiology, and pathology fellows, as well as a small number of non-clinical fellows. They were recruited via email and given three weeks in February to March 2021 to complete a seven-question survey using Google Forms online survey software. One question was added to this survey to clarify intervention design specifically related to fellows, unrelated to identification of stressors.

### Survey question

For both surveys, the first five to six items were logistical questions regarding the intervention (e.g. the best meeting time, ideal number of sessions) and are not presented in this study. The last question, analyzed in this study, was open-ended and optional: ‘What are some stressors that you face in residency/fellowship? Stressors include anything from discrimination to lack of sleep to moral distress to financial instability’. In order to maintain anonymity and encourage participation, due to the small sample size, demographic data was not collected in this survey.

### Thematic analysis

Inductive thematic analysis was conducted based on a well-accepted framework [24]. Briefly, (1) We familiarized ourselves with the data. It was downloaded from the survey software in .csv format into Microsoft Excel with each participant’s open-ended response in a single cell. The responses were divided based on complete phrases into individual data points (stressors). (2) Once data was delimited, NB coded the data, using the same codes for fellow and resident data. Using the master code list and raw data, SR independently coded the same data. The codes were compared between coders and reconciled through iterative discussion. (3) The final code list was used to determine themes, subthemes, and categories for the two separate groups, which were then reviewed and compared to the data sets to make sure that they completely encompassed the survey responses. Codes only needed to be included once to be considered when determining themes and subthemes.

### Statistical analysis

Each category was then quantified by its frequency – the percentage of respondents whose answers referenced it. The frequency that each category, subtheme, or theme was mentioned was compared between residents using a chi-square distribution test or a Fisher’s exact test, as appropriate in Microsoft Excel.

## Results

### Survey completion

Of 159 total eligible residents (39 medicine-pediatrics and 120 pediatrics), 84 (53%) completed the needs assessment, and 28 (18%) answered the final optional question. Of 180 total eligible fellows, 90 (50%) completed the needs assessment, and 38 (21%) answered the final optional question. Once delimited, there were 79 stressors identified by residents (averaging 2.8 per resident), and 105 identified by fellows (averaging 2.8 per fellow).

### Thematic analysis

Two major themes and five subthemes emerged in the data. The themes were divided into *Stressors at Home* (Table 1) and *Stressors at Work* (Table 2). Subthemes in the *Stressors at Home* category include *Difficulty Maintaining Overall Health* and *External Stressors*, both of which may also be present at work and are often affected by work, but largely occur outside of and are not exclusive to the work environment of medical training. *Stressors at Work* was divided into three subthemes: *Clinical Stressors Innate to Patient Care*, *Demanding Workload and Schedule*, and *Stressors Related to Culture of Work Environment*. While the responses of both residents and fellows all fit into the two major themes and five subthemes, there were unique categories within them that differentiated the individual groups. Of the *Stressors at Home*, there was one category unique to residents, two unique to fellows, and four shared between both groups. Of the *Stressors at Work*, one category was unique to residents, two were unique to fellows, and eight were shared between both groups.

**Table 1.** Subthemes, categories, and representative quotes corresponding to the theme, *Stressors at Home.*

Subtheme	Category for residents	Category for residents and fellows	Category for fellows	Representative quote(s)
Difficulty Maintaining Overall Health	Staying Physically Healthy			‘Access to healthy food’ (R18)
		Lack of or Poor Sleep		‘Lack of sleep or trouble sleeping’ (R5) ‘Struggling with sleep deprivation over multiple night shifts’ (F23)
		Mental Illness and Burnout		‘Anxiety’ (R1) ‘Burnt out, mental fatigue’ (F27)
External Stressors		Finances		‘Financial constraints (high debt, low income, little to no reserve to deal with emergencies)’ (R1)
		Loneliness and Social Isolation		‘Being away from family’ (R1) ‘Limited social network/support system’ (R5) ‘Lack of social interaction due to covid’ (F22)
			Childcare	‘Childcare stressors’ (F28)
			Underrepresented Group Discrimination	‘Feeling like I am taken less seriously as a female physician’ (F14) ‘Being LGBTQ’ (F27)

R-resident respondent; F: fellow respondent.

**Table 2.** Subthemes, categories, and representative quotes corresponding to the theme, *Stressors at Work.*

Subtheme	Category for residents	Category for residents and fellows	Category for fellows	Representative quote(s)
Clinical Stressors Innate to Patient Care		Moral Distress and Empathy Fatigue		‘Moral distress related to patient care scenarios’ (F5)
		Sick/Dying Patients and Complex Social Dynamics		‘Watching patients struggle with illness, death of patients’ (F33) ‘Difficult patients’ (F35)
			Clinical Uncertainty	‘Uncertainty in medicine’ (F9)
Demanding Workload and Schedule		Work-Life Balance		‘Minimal free time outside of work for normal life activities such as laundry, cleaning, groceries’ (R27) ‘Juggling personal and professional obligations’ (F24) ‘Missing out on life events for work’ (F33)
		Unusual, Variable, and Uncertain Work Schedule		‘Working nights’ (R28) ‘Unpredictable schedule’ (F10)
		High Workload and Expectations		‘Making time to study’ (R19) ‘Stress with leadership responsibilities’ (R24) ‘Balancing academic expectations with clinical responsibilities’ (F18)
Stressors Related to Culture of Work Environment	Lack of Clinical Autonomy			‘[Lack of control over] clinical situations’ (R5)
		Negative Work Environment		‘Working with other burnt-out practitioners/medical staff’ (R9) ‘Lack of acknowledgement at times’ (F4) ‘Perceived lack of respect’ (F16)
		Lack of Confidence and Feelings of Inadequacy		‘The stress of never feeling like we know enough’ (R15) ‘Imposter syndrome’ (F29)
		Career Planning and Lack of Professional Support		‘Career planning/deciding on your path’ (R5) ‘Job finding stress’ (F5) ‘Lack of mentorship’ (F20)
			Unclear Expectations	‘Nebulous expectations from different attendings’ (F20)

R: resident respondent; F: fellow respondent.

### Frequency and statistical analysis

**Figure 1.**
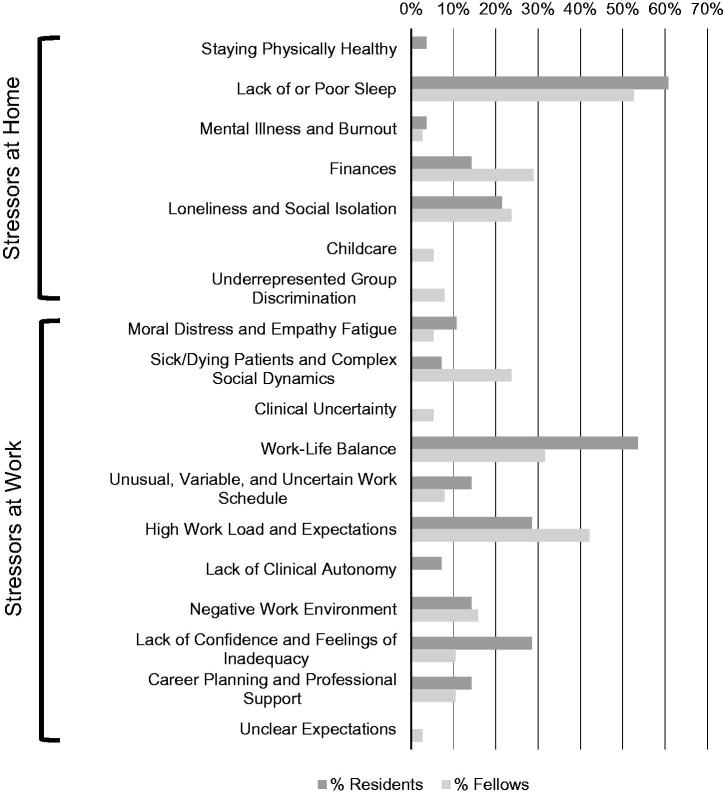
Percentage of respondents in each group (residents and fellows) who identified each category of stressor.

In addition to some categories being unique to either set of trainees, each group referenced certain stressors more frequently (Figure 1). There was no significant difference in the frequency at which various categories (*Χ*^2^(17, *N* = 184)=21.39, *p* = 0.21), subthemes (*Χ*^2^(4, *N* = 184)=7.10, *p* = 0.13), or themes (*Χ*^2^(1, *N* = 184)=0.94, *p* = 0.33) of stressors were mentioned between groups; however, there were some notable trends. Residents were more likely (54 vs. 32%) to lament their Work-Life Balance as well as their Lack of Confidence and Feelings of Inadequacy (29 vs. 11%). Whereas fellows more frequently brought up Finances (29 vs. 14%), Sick/Dying Patients and Complex Social Dynamics (24 vs 7%), and High Work Load and Expectations (42 vs. 29%).

## Discussion

In our thematic analysis of the self-reported stressors of fellows and residents at a single time point at our institution, two themes emerged: *Stressors at Home* related to those that take place outside of, though are likely affected by, the hospital, and *Stressors at Work,* those that are predominantly present in the work environment. The subthemes of *Stressors at Home* were *Difficulty Maintaining Overall Health* and *External Stressors*, representing stressors more applied by society than the workplace. The subthemes of *Stressors at Work* were *Clinical Stressors Innate to Patient Care*, that likely could not be removed, as well as those that could be mitigated, including *Demanding Workload and Schedule* and *Stressors Related to Culture of Work Environment*.

While residents’ and fellows’ stressors had common themes and subthemes, there were a few categories that were unique to fellows and some unique to residents. Within *Stressors at Home*, only residents mentioned Staying Physically Healthy as a stressor. Only fellows identified Childcare and Underrepresented Group Discrimination, and they were twice as likely to worry about Finances. Fellows tend to be older than residents, and are more likely to have children [25], which also could be associated with concern for financial stress. Regarding discrimination, a survey study by Kemper et al. [26] found that 18% of pediatric residents reported experiencing discrimination and 5.4% reported experiencing sexual harassment, while interestingly, in our study only fellows mentioned discrimination, likely due to the small sample size. The fellows who mentioned discrimination focused on mistreatment due to their gender or sexual identity, but it is important to mention that discrimination is often significantly associated with race [27] as well as gender, even in attending physicians [28], and adds additional burdens to an already difficult period of medical training. Additionally, the proportion of pediatric trainees that come from underrepresented racial and ethnic groups falls far below the representation in the general population of the United States [29], so the fact that this stressor was not mentioned does not mean that it is not present. As far as *Stressors at Work*, fellows were the only ones who identified Clinical Uncertainty, as well as Unclear Expectations, and were more likely to identify Sick/Dying Patients and Complex Social Dynamics and High Workload and Expectations, whereas residents were more concerned with a Lack of Clinical Autonomy, Lack of Confidence and Feelings of Inadequacy, and Work-Life Balance. These differences are possibly due to their respective roles on the clinical team, where fellows are given more responsibility than residents to make final decisions and have difficult discussions with families. The lack of autonomy could be associated with decreased confidence in residents that improves as they are given more responsibility as fellows. It is also important to note that these surveys were administered at two different phases of the COVID-19 pandemic. Interestingly, trainees only directly mentioned the pandemic in the context of loneliness and social isolation, and there was little difference in how frequently that stressor was present between the groups, despite the fact that the residents responded to the survey during the first 2020 summer peak, and the fellows answered in spring 2021 after vaccinations were widely available to healthcare workers, and they had had a full year to adjust to pandemic-related changes.

In our study, we were able to identify more nuanced stressors for pediatric residents than those currently in the literature, including moral distress, loneliness and social isolation, uncertain work schedule, and lack of clinical autonomy, likely because they were self-reported. No other studies have specifically identified self-reported stressors for pediatric residents. In surveys of program directors [19], pediatric residents comparing objective factors to well-being measures [21], a large study of residents comparing specialty and demographics to burnout and career satisfaction [4], and a study of pediatric residents comparing objective burnout measures and work experience [20], resident wellness has been found to be adversely associated with variety of factors. Consistent with our results, these previous studies also named sick and complex patients, work-life balance, high work load and expectations, negative work environment, lack of sleep, and financial concerns. Unlike our data, they also found that recently having made a major clinical error and being female negatively correlated with well-being in residents. It is possible that we did not identify these stressors due to the small sample size or because our pediatrics residency program is predominantly female and there have been improvements made in reducing misogyny. Notably, the themes we identified speak to a lack of control that trainees have in the work environment, over their work requirements, and in their lives outside of work as a major contributor to their stress. This lack of control is especially pronounced for residents and has long been known to be associated with resident burnout [30].

While similarities between existing literature and our data suggest generalizability of our findings, we were also able to identify lack of sleep, childcare needs, moral distress, clinical uncertainty, career planning, and unclear expectations as stressors. This suggests a level of uncertainty and ambiguity that contributes to fellowship training, as well as greater family-related needs. Stressors in pediatric subspecialty fellows have been identified in some individual subspecialties, but not broadly across all groups. At the start of their training, pediatric cardiology fellows at a single institution self-reported fears and stressors [22]. Pediatric emergency medicine and critical care fellows across the country self-reported stressors, and identified factors associated with burnout using Likert scales [5,23]. The findings in these studies were similar to ours: fellows identified stressors including mental illness and burnout, finances, underrepresented group discrimination, especially based on gender, work-life balance, unusual work schedule, high work load and expectations, negative work environment, and lack of confidence and feelings of inadequacy. Career and occupational stress, stressors identified in these surveys, broadly overlap with many of our categories, which indicates the generalizability of our results, while we were also able to capture the more nuanced stressors mentioned above.

There are limitations to our study. As the sample size for each group was approximately 30-40 respondents, it likely did not capture all stressors experienced by our trainees. Additionally, the survey question related to stressors was optional, so while >50% of residents and fellows responded to the entire survey, only about 20% responded to the last question about stressors, which may have created selection bias, affecting the overall stressors mentioned as well as their frequency in each group and the ability to detect statistically significant differences. While we acknowledge that open-ended and optional questions have lower response rates, we prioritized the potential for meaningful qualitative data and thus designed the survey this way. Another limitation is the lack of associated data collected about the survey participants, including information about demographics, year in training, and fellow subspecialty that could be used to further understand the stressors. Especially for fellows where there are only a few in each subspecialty, not collecting demographic data allowed us to maintain anonymity. The example stressors provided in the question, done so to encourage responses, may have biased answers, especially due to the frequency of lack of sleep being mentioned; but other stressors, such as work-life balance, were still reported at a high frequency, indicating that we were still able to obtain meaningful data. Our study also took place at a single, albeit large, institution, so while findings may be generalizable, many of the stressors mentioned reference the specific culture of the organization, and other programs would benefit from doing their own internal assessments. While next steps could include a similar nationwide survey, there is utility in each institution, with its own idiosyncrasies, culture, and program structure, doing its own assessment in order to direct change.

However, our study still has many strengths. First, we analyzed self-reported data from participants in order to more comprehensively capture the stressors that pediatric residents and fellows face during training. This is a significant contribution because, despite the small sample size, we were able to identify several more nuanced stressors that have not been previously reported from surveys that had respondents pick from pre-determined lists. Additionally, we directly compared pediatric residents and fellows across multiple subspecialties within the same hospital. Not only did we identify stressors that can be targeted for wellness interventions and programmatic support, but we but found that fellows and residents experience stressors unique to their stage in training. This is important for program leaders, and underscores the need for tailored, individualized interventions for residency and fellowship programs to support their trainees. Broad wellness interventions at the institutional or graduate medical education level may not target the unique and different needs of pediatric residents and fellows. Institutions should obtain input from trainees at all levels, having them serve on programming committees together, and may even consider conducting their own internal needs assessments, whether through surveys or further probing the data through interviews and focus groups.

With regards to interventions, our study identified stressors that cannot be changed versus those that can. Part of becoming a well-rounded physician includes learning how to skillfully navigate difficult conversations and challenging ethical scenarios. Programs should help trainees by specifically allocating time and resources to help them develop the necessary knowledge, attitudes and skills necessary to approach these situations with openness, maturity, and cultural humility. Successful interventions have included debriefing sessions and mindfulness programs, including Mindfulness-Based Stress Reduction [31]. Institutions will likely never be able to eliminate workplace conflict, but they can work on mitigating it as well as clarifying expectations, creating shared understanding, and providing professional support where possible. There are even known interventions, such as CREW (Civility, Respect, and Engagement at Work), that have shown to decrease incivility in the healthcare setting. Addressing incivility as defined by this study would mitigate many of the identified stressors, including discrimination, unclear expectations, and a negative work environment [32]. And it has been shown that worsening burnout associated with mistreatment due to race and gender can be ameliorated by protective systems and bystander support [28]. While adjustments to work hours have had mixed results [31], they have allowed for improved sleep, and the increase in free time could mitigate stressors including childcare, work-life balance, and the ability to manage physical health. Even if work hours are not decreased, there are creative ways to adjust schedules to allow for improved life outside of work. Lastly, better mentoring by attendings may help with career stress and build confidence in trainees, as well as improving clinical autonomy and feelings of uncertainty in complicated situations. They could even provide practical advice for how to manage lifestyle stressors and how to balance life at work with life at home. Medical training is innately difficult, and in order to care for our workforce, institutions must aim to diminish and/or eliminate the stressors that we can and give trainees the tools to cope with those that we cannot.

## Conclusions

In our survey of pediatric residents and fellows at a large children’s hospital, we identified that their self-reported stressors generally fell within the same themes and subthemes, but within the subthemes there were unique categories of stressors. There are many areas in which programs can assist their trainees with their wellness, and it is important to consider unique needs for different levels of learners when designing wellness-promoting efforts. Additionally, the stressors lend themselves to unique mitigation strategies, from working to eliminate the stressors themselves or teaching trainees coping techniques when that is not possible.

Future work should focus on further characterizing stressors, both in breadth and depth. Studies can sample larger populations, which would allow them to collect demographic to further understand the trainee groups. Additionally, in smaller settings, such as individual programs, they can conduct focus groups or interviews to gain a deeper understanding of stressors on trainees. Both of these approaches would allow programs to appropriately target wellness interventions to their trainees and hopefully improve the experience of medical training.

## Data Availability

The data that support the findings of this study are openly available in Open ICPSR at https://doi.org/10.3886/E156342V1, reference number [33].
